# Integrated care pathways and task shifting

**DOI:** 10.7448/IAS.17.4.19495

**Published:** 2014-11-02

**Authors:** Linda Panton

**Affiliations:** Regional Infectious Diseases Unit, Western General Hospital, Edinburgh, UK

## Abstract

Delivery of HIV care has evolved over the last 10 years, and nurse specialists are a driving force in developing new pathways to enhance patient care. Despite the continued rise in numbers of people living with HIV, the financial constraints on the NHS have unfortunately resulted in a reduction in service provision. Experienced nurses are integral to patient care management. They not only provide standardized care for stable patients, therefore increasing consultant capacity for the more complex medical patient, but have a degree of flexibility that allows newly diagnosed patients quick access to care and support. With a strong emphasis being placed on an integrated and collaborative multidisciplinary team approach, to ensure patients receive the same standard of care, Scotland's HIV centres follow an integrated care pathway. The nurse oversees the completion of this document and co-ordinates the pathway of care depending on the clinical need. Nurses develop and maintain necessary partnerships between primary care, specialist care, psychological services, social care and third sector support services. The nurse case load continues to expand and diversify. Stable patients may be maintained on therapy but are living with a stigmatized long-term chronic condition and rely on the nurse as a point of contact to access advice and support readily. The more chaotic and vulnerable clients with complex care needs require the nurse to co-ordinate their care, ensuring the appropriate agencies remain involved. Overseeing the transition of care to other units and tracing patients who are lost to follow up is also a necessity, as retention in care is paramount for the continued improvement in clinical outcomes. The contribution that specialist nurses make to the provision of HIV care is valuable and will continue to play a large role in the delivery of such care.

